# The Association Between METS-IR and Serum Ferritin Level in United States Female: A Cross-Sectional Study Based on NHANES

**DOI:** 10.3389/fmed.2022.925344

**Published:** 2022-06-28

**Authors:** Han Hao, Yan Chen, Ji Xiaojuan, Zhang Siqi, Chu Hailiang, Sun Xiaoxing, Wang Qikai, Xing Mingquan, Feng Jiangzhou, Ge Hongfeng

**Affiliations:** ^1^Department of Hematology, Anhui Medical University Affiliated to Bozhou People's Hospital, Bozhou, China; ^2^Department of General Practice, Wuhu City Second People‘s Hospital, Wuhu, China

**Keywords:** metabolic score for insulin resistance, insulin resistance, serum ferritin, cross-sectional studies, National Health and Nutrition Examination Survey (NHANES)

## Abstract

**Aim:**

The aim of this study was to investigate the association between the metabolic score for insulin resistance (METS-IR) and serum ferritin in females from the United States.

**Methods:**

We conducted a cross-sectional study with 4,182 participants from the National Health and Nutrition Examination Survey (NHANES). We used METS-IR and serum ferritin as the independent and dependent variables in this study and investigated the relationship by using multiple linear regression and verified the non-linear relationship with a smooth curve fit and threshold effect model.

**Results:**

There was a positive relationship between METS-IR and serum ferritin, with an effect value of (β = 0.29, 95% CI: 0.14–0.44) in a fully adjusted model adjusted for potential confounders. This positive correlation became more significant as METS-IR increased (p for trend < 0.001). Subsequent subgroup analyses showed that sensitive cohorts were those aged ≥40 years, black, and with a body mass index (BMI) < 24.9 kg/m^2^. In a smoothed curve fit analysis, the correlation between METS-IR and serum ferritin was a straight linear relationship in all participants included in this study, but when stratified by age, race, and BMI, this positive correlation in the participants who were aged ≥40 years old, other race, and had a BMI < 24.9 kg/m^2^ was non-linear.

**Conclusions:**

There was a positive association between METS-IR and serum ferritin in United States females, and this positive association was more pronounced in participants aged ≥40 years, black race and BMI < 24.9 kg/m^2^. This positive association was non-linear in the subgroups aged ≥40 years, white race and BMI < 24.9 kg/m^2^, with inflection points for METS-IR of 69.97, 67.84 and 35.84 in these respective subgroups.

## Introduction

Ferritin is the main form of protein that reflects the body's stored iron content and can be detected in blood and many tissues (1), and serum ferritin is commonly used clinically as the most important indicator of iron status. Although not a direct assessment of circulating iron levels, serum ferritin can help us to identify iron overload in a timely manner (2, 3). Iron overload can cause multi-system disorders. In the liver, for example, excess iron levels can lead to severe organ damage and eventual progression to liver failure (4), as well as an increased risk of liver cancer, and similarly it has been shown that iron overload increases the risk of heart disease and non-alcoholic liver disease (5, 6). Ferritin is an important indicator of the body's iron stores, for example alcohol intake can lead to an increase in serum ferritin (7). In addition, many diseases have an impact on serum ferritin concentrations, such as liver disease, inflammation, tumors and metabolic syndrome (8, 9).

The metabolic syndrome is an umbrella term for a complex group of diseases dependent on genetic and environmental factors, many of whose pathogenesis remains unclear, with a current prevalence of up to 39% (10, 11). Insulin resistance, an important component of the metabolic syndrome, is a key mechanism in glucolipid metabolism (12) and is strongly associated with the development and progression of many diseases. Previous studies have shown that iron overload is positively associated with reduced insulin secretion and the development of type 2 diabetes (13), but evidence from large cohort studies is still lacking. The lack of sufficient evidence for a correlation between iron overload and insulin resistance may be related to the time-consuming, expensive and complex nature of the existing reference standard for assessing the importance of insulin resistance, the hyperinsulinemic euglycemic clamp (HEC). METS-IR is a recently developed index that aims to be a practical and valid alternative biomarker for insulin resistance (IR) (14). Studies have demonstrated the diagnostic efficacy of METS-IR over other non-insulin-based insulin resistance indices (15). However, no studies correlating METS-IR with serum ferritin have yet been reported.

Therefore, we aimed to take advantage of the NHANES database with its large sample size and representative sample to investigate the relationship between METS-IR and serum ferritin. However, as NHANES now contains only a small amount of information on serum ferritin in males, to avoid gender-induced bias, the participants included in this study were limited to females.

## Methods

### Data Source

The NHANES program began in the 1960s and is based on the use of questionnaires, physical examinations, and laboratory tests to obtain demographic, dietary, physical, and laboratory information to assess the nutritional and health status of the United States citizen population. The database keeps a frequency of updates every 2 years. We obtained the original data for this study from NHANES, the National Center for Health Statistics (NCHS) Ethics Review Committee approved the NHANES survey protocol, and all participants provided written informed consent. Because the NHANES database is open to the public, ethical review of this study was exempt.

### Participants

**Figure 1:**
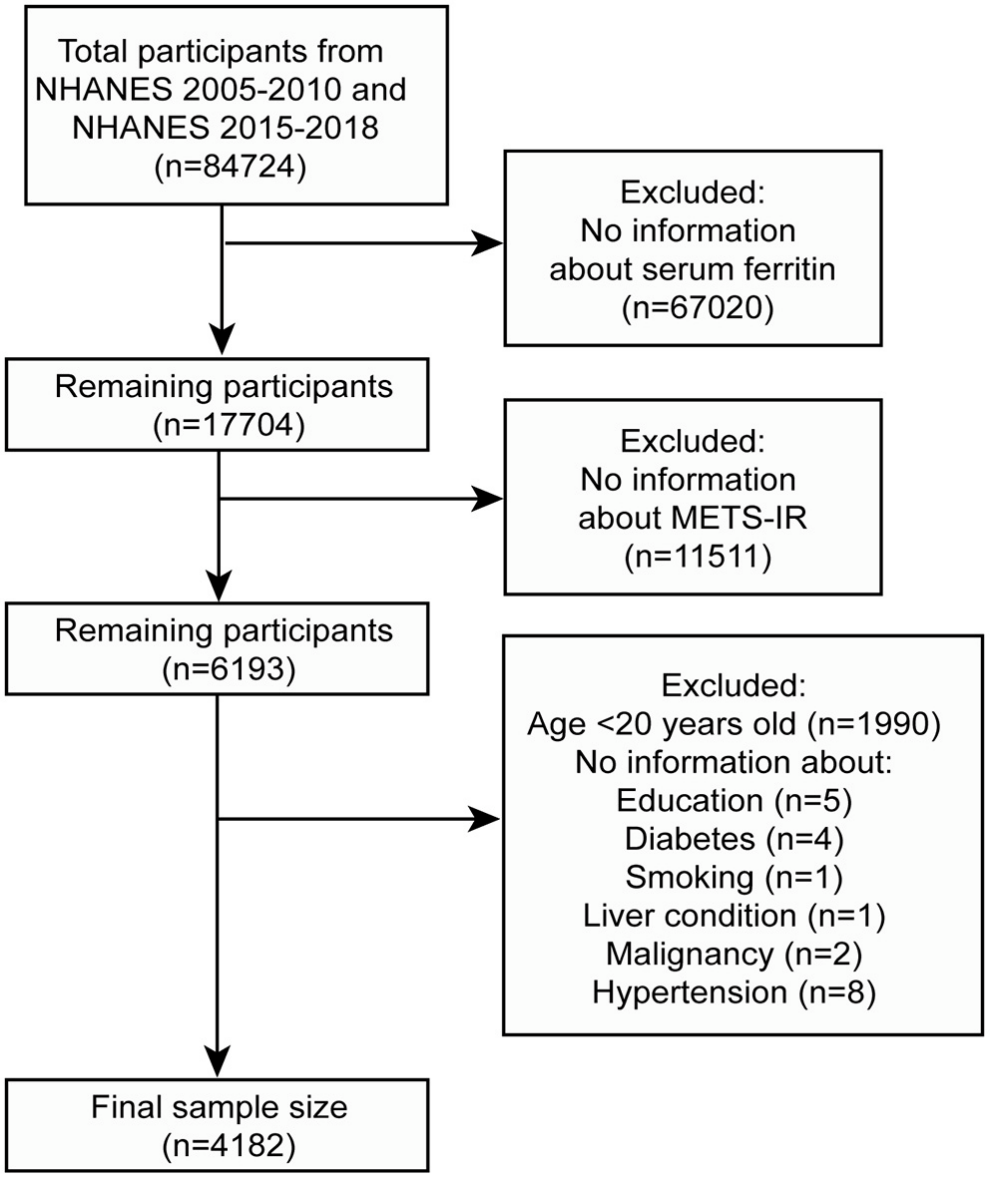
Flow chart for participants.

We first excluded male participants from NAHNES 2017–2018 (only NHANES 2017–2018 contained information on serum ferritin in males). Participants included in this study were derived from NAHENS 2005–2010 and NHANES 2015–2018, because only these two time periods contained complete information on serum ferritin. A total of 84,724 participants took part in this survey during this period. We excluded participants who had no serum ferritin information, could not calculate METS-IR (*n* = 11,511) and were younger than 20 years old (*n* = 1,990). A final total of 4,182 participants were enrolled in this study (Figure 1).

### Dependent and Independent Variables

Serum ferritin (ng/ml) as the dependent variable was derived from laboratory data. In NHANES 2004–2008, serum ferritin concentrations were measured by the Roche Tina-quant serum ferritin immunoturbidimetric method on a Hitachi 912 clinical analyser (Roche Diagnostics, Basel, Switzerland). As the Hitachi 912 clinical analyser was discontinued by the manufacturer in 2009, NHANES 2009–2010 and NHANES 2015–2018 used the Roche Elecsys 170 clinical analyser to measure serum ferritin concentrations. However, the NHANES working group converted this difference based on the Deming equation (Log10 (E170) = 0.989^*^Log10(Hitachi 912) + 0.049). METS-IR as the independent variable was not directly derived from the NHANES database. METS-IR was calculated as follows: Ln [(2 × fasting glucose (mg/dL) + fasting triglycerides (mg/dL)] × body mass index (kg/m^2^) / {Ln [high-density lipoprotein cholesterol (mg/dL)]} (14). We obtained BMI information from examination data, calculated as BMI (kg/m^2^) = weight (kg)/height (m^2^), and all other data were collected from laboratory data. Quality control of laboratory tests is available at https://wwwn.cdc.gov/nchs/nhanes/Default.aspx.

### Covariates

Following previous studies, we used additional variables as covariates in our study (6, 15, 16). The demographic information included age, race (Mexican, White, Black, Other races), ratio of family income to poverty (PIR), education level (less than high school, high school, greater than high school). Information on smoking, drinking, and disease status (diabetes, hypertension, liver conditions, history of blood transfusion, malignancy) was obtained from questionnaires, and affirmative results were obtained from the participants' positive responses to the corresponding questions. Alcohol intake and smokers were defined as having consumed alcohol at least 12 times in a year and having smoked at least 100 cigarettes in their lifetime. In addition, information on the participants' diet (energy, sugar, moisture, iron) was also collected. The patients' daily intake of total nutrients was obtained in the form of a questionnaire, calculated as the average of the sum of the values of the nutrients answered on the first and second day. Activity has a significant impact on diabetes, metabolic syndrome, and fatty liver disease (17, 18) and therefore we believe that it is also important in influencing insulin resistance. In this study, information was collected on the participants' activity level and activity was categorized as mild, moderate and vigorous based on the intensity of activity at work and recreation. Both iron overload and insulin resistance are consequences of chronic inflammation and the relationship between serum ferritin and metabolic disease could be influenced by markers of inflammation (19), we therefore obtained data on C-reactive protein (CRP) from laboratory data. All covariates were presented in Table 1.

**Table 1:** Characteristics of the participants.

**Characteristics**	**Group 1 (*n* = 2,766)**	**Group 2 (*n* = 1,416)**	***P* value**
Age (years)	33.44 ± 8.60	35.92 ± 8.62	<0.001
**Race, n (%)**			<0.001
Mexican American	951 (34.38%)	371 (26.20%)	
White	1,129 (40.82%)	671 (47.39%)	
Black	535 (19.34%)	289 (20.41%)	
Other race	151 (5.46%)	85 (6.00%)	
BMI (kg/m2)	28.43 ± 7.22	29.92 ± 8.45	<0.001
PIR	2.34 ± 1.56	2.43 ± 1.61	0.09
**Education, n (%)**			0.31
Less than high school	693 (25.05%)	327 (23.09%)	
High school	573 (20.72%)	290 (20.48%)	
More than high school	1,500 (54.23%)	799 (56.43%)	
**Smoker, n (%)**			<0.001
Yes	938 (33.91%)	590 (41.67%)	
No	1,828 (66.09%)	826 (58.33%)	
**Drinking, n (%)**			0.002
Yes	1,587 (57.38%)	891 (62.92%)	
No	884 (31.96%)	386 (27.26%)	
Unclear	295 (10.67%)	139 (9.82%)	
**Activity intensity, n (%)**			0.82
Mild	976 (35.29%)	486 (34.32%)	
Moderate	902 (32.61%)	467 (32.98%)	
Vigorous	888 (32.10%)	463 (32.70%)	
**Hypertension, n (%)**			<0.001
Yes	352 (12.73%)	264 (18.64%)	
No	2,414 (87.27%)	1,152 (81.36%)	
**Diabetes, n (%)**			<0.001
Yes	79 (2.86%)	73 (5.16%)	
No	2,670 (96.53%)	1,323 (93.43%)	
Borderline	17 (0.61%)	20 (1.41%)	
**Liver condition, n (%)**			0.04
Yes	61 (2.21%)	46 (3.25%)	
No	2,705 (97.79%)	1,370 (96.75%)	
**Malignant tumors, n (%)**			<0.001
Yes	95 (3.43%)	83 (5.86%)	
No	2,671 (96.57%)	1,333 (94.14%)	
**Total daily energy intake (kcal), n (%)**			0.103
<1,863.94	1,246 (45.05%)	687 (48.52%)	
≥1,863.94	1,099 (39.73%)	529 (37.36%)	
Unclear	421 (15.22%)	200 (14.12%)	
**Total daily sugar intake (gm), n (%)**			0.36
<112.20	1,228 (44.40%)	644 (45.48%)	
≥ 112.20	920 (33.26%)	483 (34.11%)	
Unclear	618 (22.34%)	289 (20.41%)	
**Total daily moisture intake (gm), n (%)**			<0.14
<68.69	1,267 (45.81%)	694 (49.01%)	
≥ 68.69	1,078 (38.97%)	522 (36.86%)	
Unclear	421 (15.22%)	200 (14.12%)	
**Total daily fat intake (gm), n (%)**			<0.14
<73.01	1,267 (45.81%)	694 (49.01%)	
≥73.01	1,078 (38.97%)	522 (36.86%)	
Unclear	421 (15.22%)	200 (14.12%)	
**Total daily iron intake (mg), n (%)**			
<13.95	1,376 (49.75%)	723 (51.06%)	0.58
≥13.95	969 (35.03%)	493 (34.82%)	
Unclear	421 (15.22%)	200 (14.12%)	
**Ever received a blood transfusion, n (%)**			0.16
Yes	168 (6.07%)	108 (7.63%)	
No	2,580 (93.28%)	1,298 (91.67%)	
Unclear	18 (0.65%)	10 (0.71%)	
HDL (mg/dl)	58.43 ± 16.30	55.44 ± 16.87	<0.001
FPG (mg/dl)	89.92 ± 25.62	95.00 ± 37.26	<0.001
Fasting TG (mg/dl)	123.47 ± 85.51	136.72 ± 104.48	<0.001
CRP (mg/dl)	0.44 ± 0.64	0.56 ± 0.89	<0.001
METS-IR	40.61 ± 12.59	43.99 ± 15.09	<0.001

*Mean ± SD for continuous variables: P value was calculated by weighted linear regression model*.

*% For Categorical variables: P value as calculated by weighted chi-square test*.

*BMI, body mass index; PIR, ratio of family income to poverty; FPG, Fasting glucose; HDL, High-density lipoprotein; TG, Triglycerides; CRP, C-reactive protein; METS-IR, metabolic score for insulin resistance*.

### Statistical Analysis

All data extraction and analysis were performed in R (http://www.R-project.org) and EmpowerStats (http://www.empowerstats.com). Continuous variables were expressed as means ± standard deviations, and categorical variables were expressed as percentages. In order to make the NHANES data more representative of the whole United States cohort, we used 2-year sample weights in this study. If the missing data for a continuous variable was within 10%, we would use the average of that variable instead. Otherwise, continuous variables would be grouped according to specific rules and the missing data would be set as a separate “unclear group”. If more than 10 samples were missing for a categorical variable, the missing data will be grouped separately as the “unclear group”, otherwise they would be deleted. Multiple linear regression analysis was used in the analysis to explore the relationship between METS-IR and serum ferritin. Depending on the adjustment for covariates, three models were generated. Model 1: no adjustment for covariates; Model 2: age and race were adjusted; Model 3: all covariates shown in Table 1 were adjusted. This was followed by a subgroup analysis to find more sensitive cohorts worthy of attention. Smoothed curve fitting and threshold effect models in this study were used to verify the existence of non-linear relationships between the independent and response variables. A non-linear effects model was used when the likelihood ratio (LLR) was < 0.05.

## Results

### Characteristics of Participants

A total of 4,182 participants were enrolled in this study. The mean serum ferritin was 54.04 ng/ml and participants were divided into two groups (group 1 with serum ferritin <54.04 ng/ml and group 2 with serum ferritin ≥54.04 ng/ml) based on whether their serum ferritin exceeded this mean. Group 2 had a higher METS-IR (*p* < 0.001) which was shown in Table 1.

### Association of METS-IR With Serum Ferritin

In all multiple linear regression models, a significant positive correlation was found between METS-IR and serum ferritin (Table 2). This positive correlation was present in all groups when METS-IR was grouped in quartiles, but was statistically different in the highest METS-IR group, and the trend for this positive correlation was more pronounced as METS-IR increased (*P* for trend < 0.001). What this suggested was a trend toward higher serum ferritin in participants as METS-IR increased, with this positive correlation being statistically significant once METS-IR exceeded 49.06. In subsequent subgroup analyses, this positive correlation was present across all age groups, but was more pronounced in participants aged ≥40 years (β = 0.36, 95% CI: 0.02 to 0.71). This positive association was mainly statistically different among white race (β = 0.33, 95% CI: 0.09 to 0.57), black race (β = 0.52, 95% CI: 0.19 to 0.85) and participants with a BMI ≥ 24.9 kg/m^2^ (β = 0.43, 95% CI: 0.23 to 0.63).

**Table 2:** Association between METS-IR and serum ferritin (ng/ml).

	**Model 1, β (95% CI)**	**Model 2, β (95% CI)**	**Model 3, β (95% CI)**
METS-IR	0.54 (0.41, 0.68)	0.49 (0.36, 0.63)	0.29 (0.14, 0.44)
**Quintiles of METS-IR**			
Q1 (18.91–31.62)	Reference	Reference	Reference
Q2 (31.63–38.90)	2.4 (−2.69, 7.52)	1.87 (−3.19, 6.93)	0.91 (−4.15, 5.97)
Q3 (38.91–49.05)	5.71 (0.60, 10.81)	4.98 (−0.13, 10.08)	2.54 (−2.66, 7.75)
Q4 (49.06–124.67)	16.50 (11.40, 21.61)	14.69 (9.57, 19.80)	7.10 (1.44, 12.76)
P for trend	<0.001	<0.001	<0.001
**Stratified by age (years)**			
<40	0.42 (0.28, 0.55)	0.44 (0.30, 0.58)	0.29 (0.13, 0.44)
≥40	0.66 (0.38, 0.94)	0.67 (0.38, 0.95)	0.36 (0.02, 0.71)
**Stratified by race**			
Mexican American	0.41 (0.17, 0.66)	0.34 (0.10, 0.59)	0.17 (−0.10, 0.44)
White	0.58 (0.37, 0.78)	0.51 (0.30, 0.71)	0.33 (0.09, 0.57)
Black	0.75 (0.46, 1.04)	0.67 (0.38, 0.95)	0.52 (0.19, 0.85)
Other race	0.32 (−0.20, 0.84)	0.29 (−0.23, 0.81)	−0.09 (−0.74, 0.57)
**Stratified by BMI (kg/m**^**2**^)			
<24.9	0.82 (0.09, 1.55)	0.82 (0.09, 1.55)	0.62 (−0.12, 1.37)
≥24.9	0.71 (0.53, 0.89)	0.65 (0.47, 0.83)	0.43 (0.23, 0.63)

^*^Model 1: No covariates were adjusted. Model 2: Age, race were adjusted. Model 3: All the covariates in Table 1 were adjusted.

^*^In the subgroup analysis stratified by each covariate, the model is not adjusted for the stratification variable itself.

### Smoothing Curve Fitting and Threshold Effect Analysis

**Figure 2:**
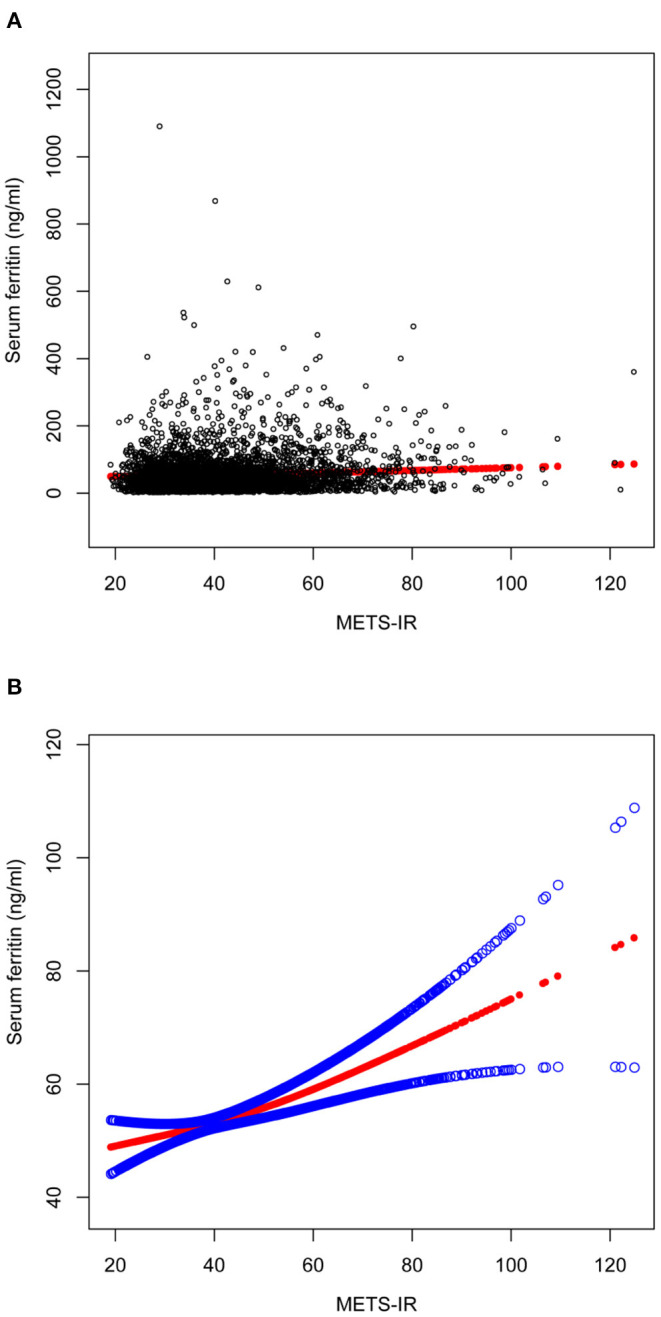
The association between METS-IR and serum ferritin. **(A)** Each black point represents a sample. **(B)** Solid red line represents the smooth curve fit between variables. Blue bands represent the 95% of confidence interval from the fit. All the covariates in Table 1 were adjusted.

**Figure 3:**
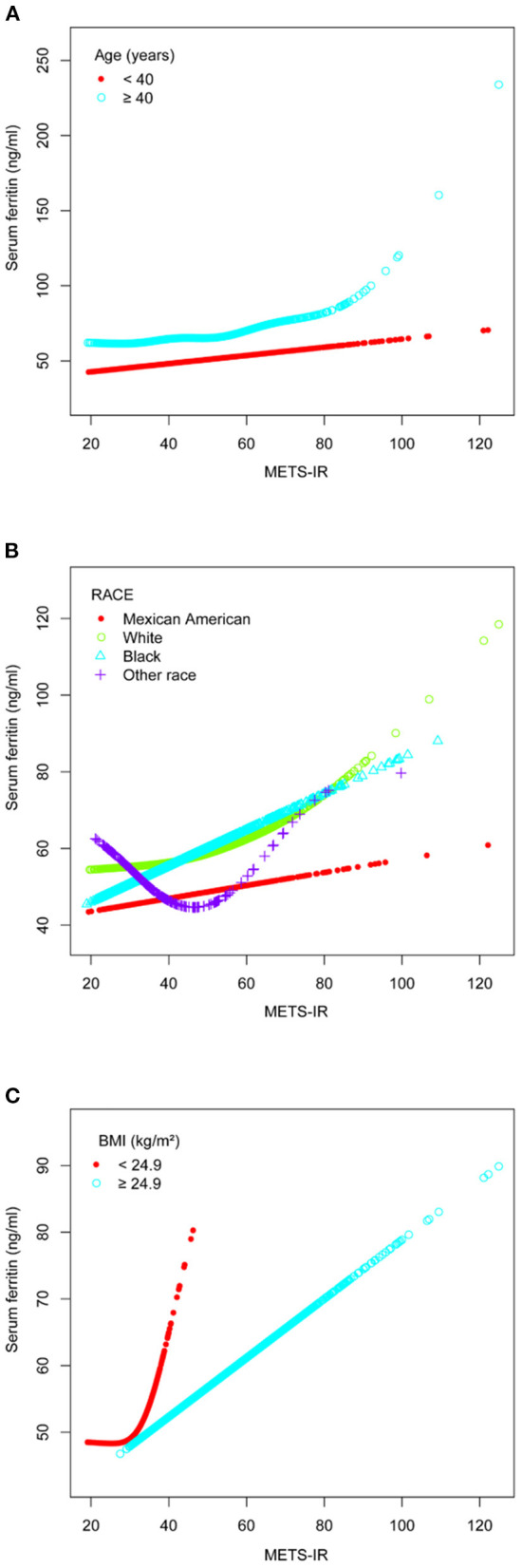
The association between METS-IR and serum ferritin. **(A)** Stratified by age (years). **(B)** Stratified by race. **(C)** Stratified by BMI (kg/m^2^). All the covariates in Table 1 were adjusted. In the subgroup analysis stratified by each covariate, the model is not adjusted for the stratification variable itself.

A smooth curve fit was performed to verify that the positive correlation between METS-IR and serum ferritin was linear (Figure 2), and further validation was done with a threshold effect model (Supplementary Table 1). To verify whether the positive correlation between METS-IR and serum ferritin was non-linear across different characteristics of the cohort, the analysis was performed by smoothing curve fitting stratified by age, race and BMI (Figure 3). The final results suggested that the METS-IR positive association with serum ferritin was curvilinear in participants aged ≥40 years, partial races (white, other races), and BMI < 24.9 kg/m^2^, for which we further performed a threshold effect analysis. Threshold effect models suggested that the positive association with serum ferritin was more pronounced in patients aged ≥40 years when METS-IR exceeded 69.97 (β = 1.84, 95% CI: 0.11 to 3.24) (Table 3). This positive correlation also requires a non-linear effects model in the white race with a METS-IR inflection point of 67.84 (β = 1.13, 95% CI: 0.05 to 2.22) (Table 4). The positive association between METS-IR and serum ferritin in the previous subgroup analysis was not statistically significant in participants with BMI < 24.9kg/m^2^, but this positive association remained statistically different when METS-IR exceeded 35.84 (β = 4.61, 95% CI: 0.42 to 8.80) (Table 5).

**Table 3:** Threshold effect analysis of METS-IR and serum ferritin (ng/ml) stratified by age (years).

**Age (years)**	** <40**	**≥40**
**Model 1**, **β** **(95% CI)**		
Linear effect model	0.29 (0.13, 0.44)	0.36 (0.02, 0.71)
**Model 2**, **β** **(95% CI)**		
Inflection point (K)	65.47	69.97
<K	0.35 (0.16, 0.53)	0.17 (−0.22, 0.56)
>K	−0.07 (−1.06, 0.23)	1.84 (0.11, 3.24)
LLR	0.205	0.034

^*^Model 1: Linear effects model; Model 2: Non-linear effects model.

^*^All the covariates in Table 1 were adjusted.

^*^In the subgroup analysis stratified by each covariate, the model is not adjusted for the stratification variable itself.

**Table 4:** Threshold effect analysis of METS-IR and serum ferritin (ng/ml) stratified by race.

**RACE**	**Mexican American**	**White**	**Black**	**Other race**
**Model 1**, **β** **(95% CI)**				
Linear effect model	0.17 (−0.10, 0.44)	0.33 (0.09, 0.57)	0.52 (0.19, 0.85)	−0.09 (−0.74, 0.57)
**Model 2**, **β** **(95% CI)**				
Inflection point (K)	28.24	67.84	46.56	37.51
<K	−1.22 (−5.10, 2.67)	0.19 (0.08, 0.47)	0.78 (0.12, 1.44)	−1.38 (−2.96, 0.20)
>K	1.42 (−2.55, 5.38)	1.13 (0.05, 2.22)	0.34 (−1.42, 0.55)	0.51 (−0.21, 3.98)
LLR	0.479	0.040	0.374	0.061

^*^Model 1: Linear effects model; Model 2: Non-linear effects model.

^*^All the covariates in Table 1 were adjusted.

^*^In the subgroup analysis stratified by each covariate, the model is not adjusted for the stratification variable itself.

**Table 5:** Threshold effect analysis of METS-IR and serum ferritin (ng/ml) stratified by BMI (kg/m^2^).

**BMI (kg/m^**2**^)**	** <24.9**	**≥24.9**
**Model 1**, **β** **(95% CI)**		
Linear effect model	0.62 (−0.12, 1.37)	0.43 (0.23, 0.63)
**Model 2**, **β** **(95% CI)**		
Inflection point (K)	35.84	33.67
<K	0.19 (−0.64, 1.03)	2.73 (−2.78, 8.23)
>K	4.61 (0.42, 8.80)	0.41 (0.21, 0.62)
LLR	0.029	0.411

^*^Model 1: Linear effects model; Model 2: Non-linear effects model.

^*^All the covariates in Table 1 were adjusted.

^*^In the subgroup analysis stratified by each covariate, the model is not adjusted for the stratification variable itself.

## Discussion

The effect of metabolic disease on serum ferritin is still controversial (20), the relationship between insulin resistance, an important component and initiator of many metabolic diseases, and ferritin remains unknown (21), and low serum ferritin levels and iron overload are undoubtedly detrimental to the body. Our study found a positive and linear correlation between METS-IR and serum ferritin in United States females. This positive association persisted whether METS-IR was used as a continuous variable or quartiles were converted to categorical variables, and also suggested that the statistical difference in this association was more pronounced at higher METS-IR. In the subgroup analysis we identified cohorts that deserve more attention, but also found that this positive correlation was also non-linear across subgroups.

No studies to date have given safe cut-off values for METS-IR, which means that higher scores suggest higher levels of insulin resistance. Age is an important factor in the development of the metabolic syndrome (22), so there is no doubt that the higher the age the greater the likelihood of insulin resistance. Combined with the results from the baseline data of the study population, the older age of participants with higher serum ferritin levels provided evidence for a subsequent subgroup analysis which suggested a more pronounced positive correlation between METS-IR and serum ferritin in participants aged >40 years.

According to the METS-IR calculation formula, it is undeniable that people with higher BMI are usually at higher risk of insulin resistance. However, the effect of BMI on insulin resistance is not absolute due to regional, environmental and genetic differences. Previous studies showed that although females in South America had a higher BMI, the incidence of type 2 diabetes was lower than in Filipino women (23). The strong association between insulin resistance and type 2 diabetes suggests that the occurrence of insulin resistance in people with lower BMI is of greater concern. Subgroup analysis in this study showed a non-linear relationship between MRTS-IR and serum ferritin in those with a BMI < 24.9 kg/m^2^, and that serum ferritin may be higher in participants after METS-IR exceeded 35.84. As we pointed out earlier, BMI is not absolute in the development of insulin resistance and it is worth considering the reasons behind lower BMI populations having higher METS-IR. Li's study showed a concomitant decrease in HDL levels with higher serum ferritin in participants after adjusting for confounding factors BMI (24), The results in Table 1 of this study also suggested that HDL was lower in participants with higher serum ferritin. It has also been shown that increased triglyceride levels lead to increased levels of free fatty acids, which can lead to insulin resistance and beta cell dysfunction (25). These studies suggested that abnormalities in lipid metabolism may play a more important role than BMI in the positive correlation between METS-IR and serum ferritin. And based on the METS-IR formula, it is certain that as METS-IR increases, participants with lower BMI have a higher risk of abnormal lipid metabolism. In summary, on the basis of the same METS-IR, dyslipidemia may be a potential mechanism for lower BMI individuals to have higher levels of serum ferritin. In addition, previous studies have shown a significant positive association between fat percentage and serum ferritin levels (26), and the strength of the evidence for assessing fat percentage using BMI alone may remain limited, particularly as individuals with a higher proportion of visceral fat are more likely to develop insulin resistance, and the use of BMI in this assessment is even more limited (27). In conclusion, non-obese female participants should also be assessed regularly for changes in METS-IR, particularly if METS-IR exceeds 35.84, with caution.

Racial differences in the occurrence of various diseases have been a topic of interest to researchers in a variety of fields. This study suggested a higher preponderance of Black in the positive correlation between METS-IR and serum ferritin. Previous studies have shown that Black Americans are less sensitive to insulin and have a higher risk of insulin resistance compared to non-Hispanic whites (28, 29). In participants with unknown exact ethnicity information, we found a non-linear relationship between METS-IR and serum ferritin. Although we did not obtain more statistically different results due to sample size limitations, studies related to the risk of developing metabolic diseases due to racial differences are still worth exploring (30).

The limitation of this study was that only females were selected as subjects, but due to female estrogen, women differ from men in fat distribution, energy metabolism and iron metabolism (31–33), which might be responsible for the higher risk of diabetes, insulin resistance and iron overload in females. In contrast, changes in estrogen have a greater impact on the development of a higher visceral fat percentage in postmenopausal women (22). The different assays for serum ferritin concentrations were also an unavoidable limitation in this study, but we have used the recommended Deming regression for conversion and NHANES officially indicated that the correlation coefficient between the converted data and the real data could reach 0.97, so that our analysis was still reliable, although we still believe that a uniform assay would have yielded more reliable results. In addition, in a cohort study of a medium-sized population, women with iron overload were found to be at significantly higher risk of developing diabetes than men (34). This evidence suggests that females may deserve more attention with regard to insulin resistance, iron overload. Furthermore, the age of the participants included in this study fluctuated between 20 and 49 years, which leads to the possibility that our findings may not apply to other age groups, and therefore follow-up cohort studies are still needed. Finally, some of the questionnaire-derived covariates included in this study may be subject to the presence of self-report bias.

## Conclusions

Our study demonstrated that female United States participants with higher serum ferritin had higher METS-IR levels and that this positive association was linear across all participants. The positive association between METS-IR and serum ferritin was more pronounced in participants aged ≥40 years, black race and BMI < 24.9 kg/m^2^. The positive association was non-linear in participants aged ≥40 years, white race and BMI < 24.9 kg/m^2^, and the trend was more pronounced in participants with these characteristics when METS-IR exceeded 69.97, 67.84 and 35.84.

## Data Availability Statement

The original contributions presented in the study are included in the article/Supplementary Material, further inquiries can be directed to the corresponding author/s.

## Author Contributions

Data analysis and manuscript writing: HH. Study design and statistical advice: YC. Manuscript editing: JX, ZS, and CH. Validation and review: SX, WQ, and XM. Quality control: FJ and GH. All authors agreed on the journal to which the article was to be submitted and agreed to take responsibility for all aspects of the work.

## Conflict of Interest

The authors declare that the research was conducted in the absence of any commercial or financial relationships that could be construed as a potential conflict of interest.

## Publisher's Note

All claims expressed in this article are solely those of the authors and do not necessarily represent those of their affiliated organizations, or those of the publisher, the editors and the reviewers. Any product that may be evaluated in this article, or claim that may be made by its manufacturer, is not guaranteed or endorsed by the publisher.
