# Supplementary material for: The Association Between METS-IR and Serum Ferritin Level in United States Female: A Cross-Sectional Study Based on NHANES
Source: Front Med (Lausanne). 2022 Jun 28;9:925344. doi: 10.3389/fmed.2022.925344 (PMC9273928; doi:10.3389/fmed.2022.925344)
Supplement: Supplementary file 1 [file Table_1.doc]

Supplementary Table 1. Threshold effect analysis of METS-IR and serum ferritin (ng/ml)

| Outcome: | Serum ferritin | P value |
| --- | --- | --- |
| Model 1, β (95% CI) |  |  |
| Linear effect model | 0.29 (0.14, 0.44) | < 0.001 |
| Model 2, β (95% CI) |  |  |
| Inflection point (K) | 52.38 |  |
| < K | 0.14 (-0.09, 0.37) | 0.044 |
| > K | 0.54 (0.22, 0.87) | < 0.001 |
| LLR | 0.078 |  |

Model 1: Linear effects model; Model 2: Non-linear effects model.

*All the covariates in Table 1 were adjusted.
